# Malaria Control Insecticide Residues in Breast Milk: The Need to Consider Infant Health Risks

**DOI:** 10.1289/ehp.0900605

**Published:** 2009-05-01

**Authors:** Hindrik Bouwman, Henrik Kylin

**Affiliations:** 1 School of Environmental Sciences and Development (Zoology), North-West University, Potchefstroom, South Africa; 2 Norwegian Institute for Air Research, Polar Environmental Centre, Tromsø, Norway; 3 Department of Aquatic Sciences and Assessment, Swedish University of Agricultural Sciences, Uppsala, Sweden

**Keywords:** DDT, IRS, pyrethroid, vector control, WHOPES

## Abstract

**Background:**

In many parts of the world, deliberate indoor residual spraying (IRS) of dwellings with insecticides to control malaria transmission remains the only viable option, thereby unintentionally but inevitably also causing exposure to inhabitants. Because mothers are exposed to insecticides via various routes, accumulated residues are transferred to infants via breast milk, in some cases exceeding recommended intake levels. Except for dichlorodiphenyltrichloroethane (DDT), safety of residues of other insecticides in breast milk has not been considered during World Health Organization Pesticide Evaluation Scheme (WHOPES) evaluations. However, very little is known of the health risks posed by these chemicals to infants who, in developing countries, breast-feed for up to 2 years.

**Objective:**

We evaluated the need for WHOPES to include breast milk as a potentially significant route of exposure and risk to infants when evaluating the risks during evaluation of IRS insecticides.

**Discussion:**

We present evidence showing that neurologic and endocrine effects are associated with pyrethroids and DDT at levels equal or below known levels in breast milk.

**Conclusions:**

Because millions of people in malaria control areas experience conditions of multiple sources and routes of exposure to any number of insecticides, even though lives are saved through malaria prevention, identification of potential infant health risks associated with insecticide residues in breast milk must be incorporated in WHOPES evaluations and in the development of appropriate risk assessment tools.

The unintentional exposure of people to sometimes unacceptably high levels of chemicals may (regrettably) be the only current and effective option available under certain circumstances. In 2004, an estimated 350–500 million people contracted malaria globally, of whom more than a million died (80–90% in Africa) [World Health Organization (WHO) 2007a; WHO and UNICEF 2005]. It is the cause of 18% of all deaths of children < 5 years of age in Africa and causes many other debilitations such as anemia, increased susceptibility to other diseases, and premature births. The WHO recommends three primary interventions for malaria control: diagnosis and treatment, insecticide-treated nets (ITN) and other materials, and indoor residual spraying (IRS) (WHO 2006b). For the foreseeable future, IRS with insecticides will remain one of the major methods with which to control malaria in many countries of Africa and elsewhere (WHO 2006b). The recommendations of insecticides are based on a process conducted by the World Health Organization Pesticide Evaluation Scheme (WHOPES) (WHO 2008), which includes the evaluation of human and environmental safety of these chemicals for use in malaria control (WHO 2006b). One of the IRS insecticides is dichlorodiphenyltrichloroethane (DDT), but 11 others are also recommended, with permethrin included as a treatment for ITNs (Table 1).

The list of IRS and ITN chemicals is dominated by pyrethroids (Table 1). The pyrethroids are residually effective for 3–6 months and therefore maintain bioavailable presence and effective concentrations on a variety of surfaces. Although pyrethroids were previously assumed to be environmentally benign, at least to human health (Barlow et al. 2001; Ray and Forshaw 2000; WHOPES 2002), a surge of recent literature on effects of pyrethroids in various mammalian models has appeared (Johri et al. 2006; Killian et al. 2007; Kolaczinski and Curtis 2004; Perry et al. 2007). It has recently been shown that pyrethroids are present at appreciable levels in breast milk, together with DDT (Bouwman et al. 2006; Sereda et al. 2009). In some individuals, the sum of analyzed pyrethroids (∑PYR) exceeded the ∑DDT (sum of DDT and metabolites). These studies indicated that the DDT was derived from its use in malaria control and the pyrethroids most likely were derived from domestic and home garden use, not malaria control.

In the early stages of infancy, human breast milk remains the best sole nutrient source for infants, despite the known presence of pollutants such as DDT, polychlorinated biphenyls, and pyrethroids (Landrigan et al. 2002; Mead 2008; Pronczuk et al. 2002). In developing countries, especially in rural areas, infants can be breast-fed (supplemented with other food) for up to 2 years (Bouwman et al. 2006). Because millions of people experience these combinations of sources and routes of pollutant uptake worldwide, it is indeed urgent to better characterize, understand, and manage the implications of such exposure, especially for infants. However, very little is known about the sources, routes of uptake, levels, effects, and risks of pollutants and current-use pesticides (CUPs; here defined as excluding DDT) in breast milk, an aspect that needs serious consideration (Anderson and Wolff 2000; Bouwman et al. 2006; Landrigan et al. 2002; Lee 2007; Pohl and Abadin 2008; Solomon and Weiss 2002).

## Objective

Our goal was to evaluate the need for WHOPES to include human breast milk as a potentially significant route of uptake of pyrethroids and other insecticides when considering risks to breast-feeding infants. We examined various routes of exposure and uptake and compared known residue levels in milk with published levels associated with neurologic and endocrine effects.

## Discussion

### Insecticide use and infant exposure scenarios in malaria control

IRS insecticides are applied indoors and under the outside rafters of dwellings subject to a number of considerations and constraints (Najera and Zaim 2002; WHO 2006c). One of these considerations relates to the required residual effectiveness of the insecticide applied to last the malaria transmission season (Table 1). It is therefore logical that active ingredients (AIs) used in IRS should be biologically available to control the mosquito vectors, but at the same time potentially also available for human uptake via various routes. These routes conceivably include dermal uptake, inhalation (dust and gas phase), and ingestion. As pointed out elsewhere, there probably exists a dynamic redistribution of applied insecticide through a continuous process of indoor sublimation, deposition, and revolatilization, as well as dust movement, necessitating a total homestead environment approach when considering exposure (Sereda et al. 2009).

Another important constraint is the general use of other insecticides in the same area (Najera and Zaim 2002) (Table 1), which must be considered for resistance management. In addition, agricultural and home garden use could also contribute to body burden and levels in breast milk (Bouwman et al. 2006; Sereda et al. 2009) (Table 2). IRS with malaria control insecticides is often not the only insecticide used in the immediate environment. Domestic and home garden use of insecticides in small containers is also general practice (Rother et al. 2008).

### Insecticide uptake

#### Air

Air, airborne dust, and inhalation exposure have been discussed in a recent surge of articles (e.g., Bateson and Schwatz 2008; Firestone et al. 2008; Kelly et al. 2007; McGraw and Waller 2009; Pohl and Abadin 2008; Rudel and Perovich 2009; Weschler and Nazaroff 2008; Williams et al. 2008). Although many articles considered conditions in developed countries, by extension they also support the notion that indoor air and airborne dust must be taken into account as sources of uptake and probable contributors to accumulation under circumstances that include IRS and indoor application of insecticides.

Very little is known about the uptake of pyrethroids and DDT under malaria control conditions from IRS, and even less is understood about uptake by infants. Inhalation is one possible route of intake, and it should be compared with intake via breast milk. Bed nets treated with pyrethroids (ITN) have been subjected to a risk assessment (Barlow et al. 2001). It was calculated that a 3-kg infant under a bed net would inhale 0.026 μg/day at a measured air concentration of 0.055 μg/m^3^. The amount inhaled was many orders of magnitude below any observed effect level for inhaled delta-methrin and was considered safe. Intake via air is therefore much less than via breast milk for any pyrethroid. Others have also reached similar conclusions (WHO 2005; WHOPES 2004) but did not consider dust, nor measure levels under actual conditions.

#### Dermal

Little is known about dermal uptake of pyrethroids (Barlow et al. 2001; Soderlund et al. 2002; Weschler and Nazaroff 2008; WHOPES 2004), and none by inhabitants under malaria control conditions. Redistribution throughout the dwelling might result in skin contact to infants crawling on floors. Another possible source is occupational exposure of the mothers working on nearby cotton fields (Rother et al. 2008; WHOPES 2004), which would explain the great variation in breast milk levels (Bouwman et al. 2006; Sereda et al. 2009).

#### Food and water

Table 2 uses data from subgroups of previous studies from KwaZulu-Natal (KZN), a province in South Africa (Bouwman et al. 2006; Sereda et al. 2009) to compare with maximum residue limits (MRLs) in food (WHO 2006a) and with ADI/TDIs (acceptable daily intake/tolerable daily intake). For some compounds, only a health-based value (equivalent to a TDI) was derived; for others, a guideline was either not deemed required or was not considered (WHO 2006a). WHO has water guidelines only for DDT (WHO 2006a), and the levels shown in Table 2 are far below the 1-μg/L guideline. Permethrin in water did not exceed the 20-μg/L health-based value. There were no guidelines for cypermethrin and cyfluthrin. For the detected compounds, water would be an unlikely significant source in this case (Sereda et al. 2009). Cypermethrin, cyfluthrin, and deltamethrin were not detected in bovine milk (Table 2). ∑DDT did not exceed the MRL, and only the maximum value for permethrin exceeded its MRL. How much of this would have transferred to breast milk is unknown. No information on pyrethroid levels in breast milk and bovine milk from other areas with malaria control could be found.

#### Breast milk

Although breast milk is also a food, it does not have a specific MRL listing or consideration (Food and Agricultural Organization 2005), nor are any similar guideline values available, leaving bovine milk MRLs as the only norm for evaluation. Using the compounds detected in breast milk from KZN and comparing this with the list of MRLs showed no milk-related MRLs for some of the compounds (Table 2). Means and maxima for cyfluthrin and ∑DDT and maxima for deltamethrin and permethrin breast milk levels exceeded their respective MRLs. Table 2 also lists calculations of infant uptake, based on 800 mL of breast milk consumed by a 5-kg infant (Bouwman et al. 2006). The MRL for ∑DDT is notably exceeded. Based on available ADIs and TDI, the mean levels of DDT and cyfluthrin found in breast milk exeeded these levels, whereas the maximum levels of delta-methrin and permethrin measured in breast milk exceeded their respective ADIs. There is no MRL or ADI for summed pyrethroids.

For an infant, highest uptake is likely to be via breast milk. Given that breast milk is a significant portion of an infant’s diet, the co-presence of pyrethroids and high levels of DDT, linked to the special circumstances of infants regarding their dependency on others and physiologic stage of development, is a strong concern regarding this route (Bouwman et al. 2006) and should be addressed. The human health consequences of DDT have recently been assessed, and enough evidence was found (based on 494 studies) to suggest a risk to human health (Eskenazi et al. 2009).

### Effects

The mini-review in the accompanying Supplemental Material (doi:10.1289/ ehp.0900605.S1 via http://dx.doi.org) deals with effects of pyrethroids on the neurologic and endocrine systems. Arguably, these systems are more significant in developing infants than in older children or adults. Only animal studies done at relevant levels (comparable with those in Table 2) and using < 1,000 μg/kg in food and some human studies were considered. Enough convincing evidence of effects (e.g., age-dependent toxicity, decrease in the density of muscarinic cholin-ergic receptors in the cerebral cortex, delayed puberty, and effects on behavior, emotionality, locomotor activity, testicular histology, anogenital distance, sperm counts, liver mass) is presented to conclude that pyrethroids and DDT are a credible threat to neurologic and endocrine systems of infants when exposed to these compounds via breast milk. Therefore, and without prejudice, we may ask whether the current suite of IRS and ITN chemicals has been assessed by WHOPES concerning infant exposure, acknowledging the protective effects attributable to reducing malaria transmission as a major positive outcome?

### WHOPES pesticides evaluation

Presently, WHOPES has a four-phase evaluation: *a*) efficacy and human and environmental safety; *b*) small-scale field trials including non-target fauna and harmful effects on operators; *c*) medium- to large-scale field evaluations that include safety; and *d*) establishing specifications for formulations (Najera and Zaim 2001). For safety, the population, operators, storage and transport, and environment are considered (Najera and Zaim 2002). The WHOPES recommendations for a number of insecticides are available (WHO 2008), but DDT and malathion have been in general use since before WHOPES became active. DDT is now undergoing a reevaluation (WHO 2007b).

The current WHOPES safety assessments protocol does not consider breast milk as a route of exposure. Some field trials included the collection of limited health information because of exposure of operators and inhabitants to the insecticides, based mainly on questionnaires or surveys during or following IRS application or ITN use. Few of these concerned children, and none considered infants. Therefore, as far as we are aware, WHOPES considerations have not included CUPs in breast milk or in any other public health use.

## Conclusions

We can confidently conclude that infants under malaria control conditions are exposed to combinations of chemicals that would have deleterious effects if the intakes were high enough. Table 2 shows that the intakes do exceed acceptable levels of intake. The possible resultant toxicity would be attributable to either a single compound or combinations of several that could act additively, antagonistically, independently, or possibly synergistically. Critical windows of exposure also need to be considered [see Supplemental Material (doi:10.1289/ehp.0900605.S1)]. The health effects might be transient, reversible, latent, and/or permanent, and might also be subtle and not readily attributable. Adding to the problem, IRS and ITNs also effectively reduce morbidity and mortality of malaria, resulting in a paradox that is a characteristic of many situations where risks and positive outcomes need to be measured and balanced.

Given that breast milk is a major and important source of food for infants under malaria control conditions, the clear concerns about health impacts of CUPs on neurologic and endocrine systems (among others not considered here), and the susceptibility of developing infants, it is obvious that breast milk as a vector should be considered in risk assessment. Acknowledging that prenatal exposure also has serious implications, it is during the breast-feeding period that the infant probably gets exposed to the highest lifetime concentration of insecticides (excluding occupational exposure) via a variety of routes [judging from DDT levels in blood (Bouwman et al. 1992)]. Infants and children are recognized as a special risk category for numerous well-established reasons (WHO 2006d), and we need to take heed when metrics such as TDIs, ADIs, and MRLs are exceeded.

Even though it may be argued that exposure to IRS residues is the only concern for WHOPES and that only individual IRS chemicals must be considered, the situation and practice in many areas with IRS is to switch AIs between seasons. Because DDT is so persistent, co-exposure to multiple AIs must be taken into account with any risk assessment. In addition, given the close association of many rural communities with their environments, home gardens, and adjacent agriculture (Rother et al. 2008), as well as the obvious requirement of coordinating AI selection with agriculture for vector resistance management (which seldom happens, in our experience), exposure to multiple AIs is inevitable. WHOPES should therefore include multiple chemicals, sources, and routes in their risk assessments.

The argument that malaria kills but deaths are not likely attributable to AIs under normal malaria control conditions does not reduce the responsibility to ascertain the risks posed by insecticides and delve deeper into how these risks can be mitigated. Assuming from the above that there is a health burden due to IRS AIs, however small it may be compared with death, it is likely to impose a lifelong (and possibly even transgenerational) disability, handicap, and burden on individuals and society. Malaria control cannot be halted because of these concerns. Therefore, new, safer, and alternative ways of controlling malaria should be pursued, and fortunately this is happening on many fronts. At the same time, however, risk assessments of current and potentially new AIs with regard to infant health should take into account breast milk as an exposure route. This would provide not only a way of investigating risk reduction measures on many levels, but also criteria against which to assess reduction of the impact on infant health.

This raises the question regarding the availability of tools to predict and assess infant health risk due to exposure to multiple chemicals relevant under malaria control conditions. Multiple exposure scenarios are relevant to other situations, and tools being developed for this purpose (e.g., Cedergreen et al. 2008; Clewell and Gearhardt 2002; Firestone et al. 2008; McKinlay et al. 2008; Pohl and Abadin 2008; Timchalk and Poet 2008; Trapp et al. 2008) may be applicable if situations that include malaria control scenarios are taken into account during their development. Tools that model lactational transfer of compounds will also be useful (Verner et al. 2008). It is therefore hoped that current initiatives will consider malaria control situations. In most cases, such scenarios will also accommodate other public safety chemical exposures.

The eminently practical approach and effective use of chemicals to prevent mortality and morbidity from malaria is acceptable practice (where other methods do not work) and can be improved on. However, adding infant health and exposure via breast milk to the existing set of WHOPES safety considerations of IRS chemicals will further improve safety and operational guidelines and indicate potential risk reduction interventions when exposure to chemicals is inevitable.

## Figures and Tables

**Table 1:** Insecticides recommended by WHO, with associated parameters.

Insecticide	Type	Use	IRS dosage (g/m^2^)	Maximum applied (g) per average dwelling at 42 m^2^	ITN dosage (g/m^2^)	Maximum applied (g) per average net at 15 m^2^	IRS residual effectiveness (months)	WHO hazard classification	Known recent uses in KZN province, South Africa	Assessments by WHOPES since 1997
Alpha-cypermethrin	Pyrethroid	IRS/ITN	0.02–0.03	1.26	0.02–0.04	0.6	4–6	II		1998 IRS, ITN; 2007 ITN
Bendiocarb	Carbamate	IRS	0.1–0.4	16.8			2–6	II		
Bifenthrin	Pyrethroid	IRS	0.025–0.050	2.1			3–6	II		2001 IRS
Cyfluthrin	Pyrethroid	IRS/ITN	0.02–0.05	2.1	0.05	0.75	3–6	II		1998 ITN, IRS
DDT	Organochlorine	IRS	1–2	84			> 6	II	IRS (annually)	Currently under reevaluation
Deltamethrin	Pyrethroid	IRS/ITN	0.020–0.025	1	0.015–0.025	0.38	3–6	II	Agriculture/home	1997, 1999, 2001, 2004, 2007 ITN; 2002 IRS
Etofenprox	Pyrethroid	IRS/ITN	0.1–0.3	12.6	0.2	3	3–6	II		1997 IRS, ITN; 1999, 2001 ITN
Fenitrothion	Organophosphate	IRS	2	84			2	II		
Lamda-cyhalothrin	Pyrethroid	IRS/ITN	0.02–0.03	1.26	0.01–0.015	0.23	3–6	II		2001 ITN; 2007 IRS
Malathion	Organophosphate	IRS	2	84			2	III		
Permethrin	Pyrethroid	ITN			0.2–0.5	0.75		II	Agriculture/home	
Primiphos-methyl	Organophosphate	IRS	1–2	84			1–2	III		
Propoxur	Carbamate	IRS	1–2	84			1–2	II		

**Table 2:** Residue levels of WHO-recommended insecticide in water and milk compared with available MRLs, ADIs, and TDIs.

Insecticide	Levels in drinking water from KZN [min/mean/max (μg/L), %pos]^a^	WHO water guideline (μg/L)	Levels in bovine milk from KZN [min/mean/max (μg/kg mf), %pos]^a^	MRL bovine milk (μg/kg mf)	Levels in breast milk from KZN [min/mean/max μg/L wm), %pos]	Factors breast milk exceeding MRL for bovine milk (min/mean/max)	ADI/TDI (μg/kg bw)	Infant daily intake from breast milk (μg/kg bw) (min/mean/max)	Factors ADI/TDI exceeded (min/mean/max)
Alpha-cypermethrin	ND/0.028/0.034, 8 (*n* = 28)	NG	ND (*n* = 10)	50	ND/4.2/28, 15 (*n* = 52)^b^	ND/0.08/0.56	20	ND/0.67/4.5	ND/0.03/0.22
Bendiocarb	NA	NC	NA	No MRL	NA		4		
Bifenthrin	NA	NC	NA	50	NA		20		
Cyfluthrin	ND/0.0095/0.015, 12 (*n* = 28)	NC	ND (*n* = 10)	10	ND/42/459, 25 (*n* = 52)^b^	ND/4.2/45.9	20	ND/6.7/73	ND/0.34/3.8
Total DDT	ND/0.0065/0.021, 48 (*n* = 28)	1	0.74/4.4/13, 100 (*n* = 10)	20	70/308/725, 100 (*n* = 13)^a^	3.5/15/36	10	11/49/116	1.1/4.9/11.6
Deltamethrin	ND (*n* = 28)	NG	ND (*n* = 10)	50	ND/8.4/83, 31 (*n* = 52)^b^	ND/0.17/1.7	10	ND/1.3/13	ND/0.13/1.3
Etofenprox	NA	NC	NA	No MRL	NA		30		
Fenitrothion	NA	NG (8)	NA	2	NA		5		
Lamda-cyhalothrin	NA	NC	NA	No MRL	NA		5		
Malathion	NA	NG (900)	NA	No MRL	NA		300		
Permethrin	ND/0.066/0.067, 8 (*n* = 28)	NG (20)	ND/64/118, 60 (*n* = 10)	100	ND/57/113, 77 (*n* = 13)^a^	ND/0.57/1.13	50	ND/9.1/18.1	ND/0.18/0.36
Primiphos-methyl	NA	NC	NA	10	NA		5		
Propoxur	NA	NG	NA	No MRL	NA		20		

Abbreviations: bw, body weight; max, maximum; mf, milk fat; min, minimum; MRL, maximum residue limit for whole bovine milk; NA, not analyzed; NC, not considered; ND, not detected; NG, no guideline for water—judged not needed (value in parentheses refer to health-based value derived from ADI); %pos, percentage positive; wm, whole milk.

a Data from Sereda et al. (2009).

b Data from Bouwman et al. (2006).

## References

[b1-ehp-117-1477] Anderson HA, Wolff MS (2000). Environmental contaminants in human milk. J Expo Sci Environ Epidemiol.

[b2-ehp-117-1477] Barlow SM, Sullivan FM, Lines J (2001). Risk assessment of the use of deltamethrin on bednets for the prevention of malaria. Food Chem Toxicol.

[b3-ehp-117-1477] Bateson TF, Schwatz J (2008). Children’s response to air pollutants. J Toxicol Environ Health A.

[b4-ehp-117-1477] Bouwman H, Becker PJ, Cooppan RM, Reinecke AJ (1992). Transfer of DDT used in malaria control to infants via breast milk. Bull WHO.

[b5-ehp-117-1477] Bouwman H, Sereda B, Meinhardt HM (2006). Simultaneous presence of DDT and pyrethroid residues in human breast milk from a malaria endemic area in South Africa. Environ Pollut.

[b6-ehp-117-1477] Cedergreen N, Christenses AM, Kamper A, Kudsk P, Mathiassen SK, Streibig JC (2008). A review of independent action compared to concentration addition as reference models for mixtures of compounds with different molecular target sites. Environ Toxicol Chem.

[b7-ehp-117-1477] Clewell RA, Gearhardt JM (2002). Pharmacokinetics of toxic chemicals in breast milk: use of PBPK models to predict exposure. Environ Health Perspect.

[b8-ehp-117-1477] Eskenazi B, Chevrier J, Goldman Rosas L, Anderson AA, Bornman MS, Bouwman H (2009). The Pine River Statement: human health consequences of DDT use. Environ Health Perspect.

[b9-ehp-117-1477] Firestone M, Sonawane B, Barone S, Salmon AG, Brown JP, Hattis D (2008). Occurrence of endocrine-disrupting potential new approaches for children’s inhalation risk assessment. J Toxicol Environ Health A.

[b10-ehp-117-1477] Food and Agricultural Organization (2005). Codex Alimentarius Homepage.

[b11-ehp-117-1477] Johri A, Yadav S, Singh RL, Dhawan A, Ali M, Parmar D (2006). Long lasting effects of prenatal exposure to deltamethrin on cerebral and hepatic cytochrome P450s and behavioral activity in rat offspring. Eur J Pharmacol.

[b12-ehp-117-1477] Kelly BC, Ikonomou MG, Blair JD, Morin AE, Gobas FAPC (2007). Food web-specific biomagnification of persistent organic pollutants. Science.

[b13-ehp-117-1477] Killian E, Delport R, Bornman MS, De Jager C (2007). Simultaneous exposure to low concentrations of dichlorodiphenyltrichloroethane, deltamethrin, nonylphenyl and phytoestrogens has negative effects on the reproductive parameters in male Sprague-Dawley rats. Andrologia.

[b14-ehp-117-1477] Kolaczinski JH, Curtis CF (2004). Chronic illness as a result of low-level exposure to synthetic pyrethroid insecticides: a review of the debate. Food Chem Toxicol.

[b15-ehp-117-1477] Landrigan PJ, Sonawane B, Mattison D, McCally M, Garg A (2002). Chemical contaminants in breast milk and their impact on children’s health: an overview. Environ Health Perspect.

[b16-ehp-117-1477] Lee GL (2007). Lactation and drugs. Paediatr Child Health.

[b17-ehp-117-1477] McGraw JE, Waller DP (2009). Fish ingestion and congener specific polychlorinated biphenyl and *p,p′*-dichloro-diphenyldichloroethylene sperm concentrations in a Great Lakes cohort of pregnant African American women. Environ Int.

[b18-ehp-117-1477] McKinlay R, Plant JA, Bell JNB, Voulvoulis N (2008). Calculating human exposure to endocrine disrupting pesticides via agricultural and non-agricultural exposure routes. Sci Total Environ.

[b19-ehp-117-1477] Mead MN (2008). Contaminants in human milk: weighing the risks against the benefits of breastfeeding. Environ Health Perspect.

[b20-ehp-117-1477] Najera JA, Zaim M (2001). Malaria Vector Control: Insecticides for Indoor Residual Spraying. WHO Pesticide Evaluation Scheme.

[b21-ehp-117-1477] Najera JA, Zaim M (2002). Malaria Vector Control: Decision Making Criteria and Procedures for Judicious Use of Insecticides. WHO Pesticide Evaluation Scheme.

[b22-ehp-117-1477] Perry MJ, Venners SA, Barr DB, Xu X (2007). Environmental pyrethroid and organophosphorous insecticide exposure and sperm concentration. Reprod Toxicol.

[b23-ehp-117-1477] Pohl HR, Abadin HG (2008). Chemical mixtures: evaluation of risk for child-specific exposures in a multi-stressor environment. Toxicol Appl Pharmacol.

[b24-ehp-117-1477] Pronczuk J, Akre J, Moy G, Vallenas C (2002). Global perspectives in breast milk contamination: infectious and toxic hazards. Environ Health Perspect.

[b25-ehp-117-1477] Ray DE, Forshaw PJ (2000). Pyrethroid insecticides: poisoning syndromes, synergies and therapy. Clin Toxicol.

[b26-ehp-117-1477] Rother H-A, Hall RH, London L (2008). Pesticide use among emerging farmers in South Africa: contributing factors and stakeholders perspectives. Dev South Afr.

[b27-ehp-117-1477] Rudel RA, Perovich LJ (2009). Endocrine disrupting chemicals in indoor and outdoor air. Atmos Environ.

[b28-ehp-117-1477] Sereda B, Bouwman H, Kylin H (2009). Comparing water, bovine milk, and indoor residual spraying as possible sources of DDT and pyrethroid residues in breast milk. J Toxicol Environ Health A.

[b29-ehp-117-1477] Soderlund DM, Clark JM, Sheets LP, Mullin LS, Piccirillo VJ, Sargent D (2002). Mechanisms of pyrethroid neuro-toxicity: implications for cumulative risk assessment. Toxicology.

[b30-ehp-117-1477] Solomon GM, Weiss PM (2002). Chemical contaminants in breast milk: time trends and regional variability. Environ Health Perspect.

[b31-ehp-117-1477] Timchalk C, Poet TS (2008). Development of a physiologically based pharmacokinetic and pharmacodynamic model to determine dosimetry and cholinesterase inhibition for a binary mixture of chlorpyrifos and diazinon in the rat. Neurotoxicology.

[b32-ehp-117-1477] Trapp ST, Bomholtz LM, Legind CN (2008). Coupled mother-child model for bioaacumulation of POPs in nursing infants. Environ Pollut.

[b33-ehp-117-1477] Verner MA, Ayotte P, Muckle G, Charbonneau M, Haddad S (2009). A physiologically based pharmacokinetic model for the assessment of infant exposure to persistent organic pollutants in epidemiologic studies. Environ Health Perspect.

[b34-ehp-117-1477] Weschler CJ, Nazaroff WW (2008). Semivolatile organic compounds in indoor environments. Atmos Environ.

[b35-ehp-117-1477] WHO (2005). Safety of Pyrethroids for Public Health Use.

[b36-ehp-117-1477] WHO (2006a). Guidelines for Drinking-Water Quality. First Addendum to Third Edition, Vol 1—Recommendations.

[b37-ehp-117-1477] WHO (2006b). Indoor Residual Spraying: Use of Indoor Residual Spraying for Scaling up Global Malaria Control and Elimination.

[b38-ehp-117-1477] WHO (2006c). Pesticides and Their Application for the Control of Vectors of Public Health Importance.

[b39-ehp-117-1477] WHO (2006d). Principles for Evaluating Health Risks in Children Associated with Exposure to Chemicals. Environmental Health Criteria 237.

[b40-ehp-117-1477] WHO (2007a). Malaria Elimination: A Field Manual for Low and Moderate Endemic Countries.

[b41-ehp-117-1477] WHO (2007b). The Use of DDT in Malaria Vector Control: WHO Position Statement.

[b42-ehp-117-1477] WHO (World Health Organization) (2008). WHO Pesticides Evaluation Scheme.

[b43-ehp-117-1477] WHO and UNICEF (2005). World Malaria Report 2005.

[b44-ehp-117-1477] WHOPES (WHO Pesticide Evaluation Scheme) (2002). Report of the Sixth WHOPES Working Group Meeting. Deltamethrin 25% WG & WP and Agnique MMF.

[b45-ehp-117-1477] WHOPES (WHO Pesticide Evaluation Scheme) (2004). A Generic Risk Assessment Model for Insecticide Treatment and Subsequent Use of Mosquito Nets.

[b46-ehp-117-1477] Williams MK, Rundle A, Holmes D, Reyes M, Hoepner LA, Barr DB (2008). Changes in pest infestation levels, self-reported pesticide use, and permethrin exposure during pregnancy and after 2000–2001 U.S. Environmental Protection Agency restriction on organophosphates. Environ Health Perspect.

